# Irregular meal pattern and later sleep midpoint are associated with increased BMI *z*-score and waist–height ratio during early adolescence

**DOI:** 10.3389/fped.2024.1321024

**Published:** 2024-11-15

**Authors:** Sohvi Lommi, Elina Engberg, Aku-Ville Lehtimäki, Reetta Lehto, Heli Viljakainen

**Affiliations:** ^1^ Folkhälsan Research Center, Helsinki, Finland; ^2^ Faculty of Medicine, University of Helsinki, Helsinki, Finland; ^3^ Department of Psychology and Logopedics, University of Helsinki, Helsinki, Finland; ^4^ Department of Food and Nutrition, University of Helsinki, Helsinki, Finland

**Keywords:** obesity, exercise, screen time, night, diet, youth

## Abstract

**Background:**

Rapid gains in adiposity may have more adverse health implications in later life compared with having stable adiposity throughout childhood and adolescence. A knowledge gap concerns concomitant health behaviors contributing to adiposity gain among adolescents.

**Objectives:**

We investigated the associations of health behaviors relating to dietary habits, sleep, physical activity (PA), and screen time with an increase in body mass index *z*-score (BMIz) and waist–height ratio (WHtR) during adolescence.

**Methods:**

We included 4,785 adolescents (53% of girls) aged 11.1 (SD 0.8) years at baseline and followed them for 3 years. We clustered them into decreased, stable, and increased BMIz and WHtR categories using the K-means clustering method. Using Cox regression, we computed hazard ratios (HR) with 95% CI for the associations of self-reported health behaviors (dietary habits, physical activity, sleep midpoint, and sedentary digital media use) with belonging to an increased BMIz or WHtR group. In a subsample (*n* = 3,840), we ran a sensitivity analysis considering puberty status as an additional covariate.

**Results:**

Later sleep midpoint (having later midpoint of sleep between bedtime and waking time) and irregular meal pattern (not eating lunch and dinner every school day) predicted increased BMIz (HR 1.26 [95% CI 1.13–1.41] and 1.23 [1.08–1.39], respectively) and WHtR (1.23 [1.09–1.39] and 1.18 [1.02–1.36], respectively) over the follow-up period, after adjusting for other health behaviors. Associations remained after considering puberty status as a covariate.

**Conclusions:**

Early bedtime with adequate sleep duration and regular meal pattern should be encouraged to prevent adiposity gain during early adolescence.

## 1 Introduction

Pediatric obesity remains a public health challenge yet to be resolved; every third child in Europe and in the United States and nearly every fifth child worldwide is estimated to live with overweight or obesity (1, 2). Childhood obesity increases the risk of adulthood morbidity, such as cardiometabolic diseases (2), but alarmingly, it is also associated with conditions such as non-alcoholic fatty liver disease and asthma already in childhood (2–4). Adolescence is a plausible period for excess weight gain (5), and excess weight tends to be tracked to adulthood (6), highlighting the need for early prevention.

Childhood obesity is typically caused by a positive energy balance induced by an obesogenic environment in combination with a genetic predisposition for weight gain (2). Behavioral risk factors, such as unhealthy dietary habits, insufficient physical activity (PA), inadequate or poor sleep, and high amounts of sedentary behaviors have been recognized as key determinants of childhood obesity (6, 7). Still, the associations appear complex, and health behaviors are frequently interrelated. For example, unfavorable sleep habits such as short sleep duration and delayed sleep timing have been associated with weight gain, but the associations may be interrelated; moreover, poor sleep can affect other health behaviors such as eating habits and lead to poor dietary quality (8–10). High screen time may impair sleep and reduce the amount of physical activity (11). Indeed, unhealthy lifestyle behaviors may depend on a plethora of multifaceted and interconnected non-modifiable factors (i.e., biological, sociodemographic, and cultural) and modifiable (i.e., behavioral) risk factors (6), making obesity prevention challenging. Thus, gaining a deeper understanding of the modifiable risk factors in relation to weight gain in youth might contribute to new preventive strategies for obesity (12).

Rapid adiposity gain in early life may pose health threats in adulthood (13), whereas a stable BMI, whether low or high, throughout childhood and adolescence may be more beneficial in regard to later adverse cardiometabolic outcomes (14, 15). According to a recent study that followed youth from early school age to young adulthood, those participants with normal weight who became overweight or obese had more abdominal fat and worse blood pressure, insulin, and cholesterol levels compared with those who had a stable BMI despite overweight or obesity (14). Thus, determinants of rapid adiposity gain might provide new insights into the research of childhood obesity. Therefore, the aim of this study is to prospectively investigate the associations of health behaviors (dietary habits, PA, sleep, and sedentary digital media use) with an increase in adiposity indicators [BMI *z*-score (BMIz) and waist–height ratio (WHtR)] over a 3-year follow-up in a large sample of Finnish adolescents.

## 2 Material and methods

### 2.1 Study design and participants

The present study utilized baseline and follow-up data from the Finnish Health in Teens (Fin-HIT) cohort study. At baseline during 2011­–2014, 11,407 adolescents at the age of 9–12 years were recruited from 44 municipalities in Finland. The study was carried out mainly in a school setting without specific exclusion criteria (16). The first follow-up data collection was conducted during 2015–2016. Informed written consent was obtained from all adolescents and from one of their caregivers/parents. The study protocol was approved by the Coordinating Ethics Committee of the Hospital District of Helsinki and Uusimaa, Finland (Nr: 169/13/03/00/10). In the present study, adolescents with data on sex, age, maternal socioeconomic status (SES), health behaviors (dietary habits, PA, sleep, and sedentary digital media use), and baseline and follow-up measures of BMIz and WHtR were included (*n* = 4,785).

### 2.2 Measurements

#### 2.2.1 Anthropometrics

Weight, height, and waist circumference were measured by trained research staff at baseline, while at follow-up, parents were instructed to measure and report their children's measurements. Home measures were deemed sufficiently accurate (17). BMI was calculated as body weight (kg)/height squared (m^2^), based on which BMIz was calculated using the age- and sex-specific reference values of the International Obesity Task Force criteria (IOTF) (18). WHtR was calculated as waist circumference (cm) divided by height (cm). Age- and sex-standardized BMIz along WHtR—which is unaffected by age and sex—are universally accepted measures for adiposity for individuals under 18 years of age and correlate with measured body fat percentage or content longitudinally (19).

We used the K-means clustering method (20) to divide the participants into three subgroups based on the similarity of changes in anthropometric measures (*Δ*BMIz or *Δ*WHtR**)**. This clustering method allows the partitioning of an individual into one cluster only. The subgroups of BMIz were as follows: (1) *Δ*BMIz ranging from −3.74 to −0.31 (decreased BMIz), (2) *Δ*BMIz ranging from −0.31 to 0.39 (stable BMIz), and (3) *Δ*BMIz ranging from 0.39 to 2.49 (increased BMIz). Similarly, we classified the participants into (1) change in WHtR ranging from −0.14 to −0.02 (decreased WHtR), (2) change in WHtR ranging from −0.02 to 0.02 (stable WHtR), and (3) change in WHtR ranging from 0.02 to 0.17 (increased WHtR).

To present baseline and follow-up characteristics of the sample, we additionally categorized the participants into thin, normal weight, overweight, or obese according to age- and sex-specific cutoffs of the IOTF (18) and into with or without central obesity based on their WHtR. A WHtR of <0.5 was considered without central obesity, and others as with central obesity.

#### 2.2.2 Health behaviors at baseline

##### 2.2.2.1 Dietary habits

Three variables on dietary habits were used: (1) dietary pattern, (2) regularity of breakfast, and (3) regularity of lunch and dinner. We obtained information on food consumption with a 16-item food frequency questionnaire (FFQ) (Supplementary Material) enquiring about food consumption during the preceding month, adapted from the FFQ used in the WHO Health Behavior in School-Aged Children HBSC study (21). The frequency of food consumption was self-reported and assessed by a seven-point scale varying from 0 (“not consumed”) to 6 (“consumed several times per day”). Three dietary patterns called “healthy eaters,” “unhealthy eaters,” and “fruit and vegetable avoiders” were identified and defined in the Fin-HIT cohort as described elsewhere (22). Healthy eaters consumed most often dark bread, fresh vegetables, fruits, and berries compared with the other groups. Fruit and vegetable avoiders had the lowest consumption frequency of fresh vegetables, fruits, and berries. Unhealthy eaters consumed sweet pastries, biscuits or cookies, ice cream, sugary juice drinks, fast food (hamburgers or hot dogs), and salty snacks most frequently. In addition, the participants reported the frequency of main meals (breakfast, school lunch, and dinner) during school weeks by answering the question “How often do you usually eat the following meals during a school week?” on a six-point scale ranging from “rarely or never” to “five days a week.” We have previously defined breakfast pattern (regularity of breakfast) and meal pattern (regularity of school lunch and dinner) (22). Eating breakfast 5 days a school week was considered a regular breakfast pattern and a frequency of less than five was considered an irregular pattern. Similar coding was utilized for meal pattern; thus, eating both school lunch and dinner 5 days a week was considered regular, and less than that as irregular.

##### 2.2.2.2 Sleep midpoint

Sleep-related questions were adapted from the HBCS study's questionnaire for 13- and 15- year-olds with minor modifications (23). Briefly, the usual bedtimes and waking hours on school days and non-school days were asked with four separate questions with multiple predefined answer options each varying 0.5 h as described elsewhere (24). Based on the responses, we calculated sleep duration (h/d) for school days and non-school days. To examine sleep timing, sleep midpoint (middle time point between bedtime and wake-up time) was calculated. Here, we used a weighted weekly mean sleep midpoint to illustrate the usual timing of the sleep, calculated as *([sleep midpoint on school days (h:mm)×5]* *+* *[sleep midpoint on non-school days (h:mm) × 2])/7*. In a similar manner, we calculated the weighted sleep duration and weighted bedtime to show these values in the descriptive tables.

##### 2.2.2.3 Physical activity

The participants reported their leisure-time PA by answering the following question adapted from another Finnish school-based study (25): “How many hours a week do you normally exercise or do sports during your free time? Include all the exercise you do in a club or team and any exercise by yourself, with family or friends. Do not count any exercise at school or on the way to school.” The response alternatives ranged from (1) “an hour a week or less” to (10) “around 10 hours a week.” These were used to indicate hours of PA/week. Those who in a previous question reported to never exercise in their free time were assigned a value of 0.

##### 2.2.2.4 Sedentary digital media use

Leisure-time digital media use was assessed with questions adapted from the HBSC study (26). We assessed TV viewing with the question: “How many hours a day during your free time do you normally watch TV, videos or DVDs? By TV, we mean programs that can be watched on TV as well as on a computer.” Further, we assessed computer use with the question: “How many hours a day during your free time do you normally use a computer, e.g., spend time on the Internet, chat or play computer or TV games sitting down (e.g., PlayStation, Xbox)?” We asked the questions about TV viewing and computer use separately for school days and for days off. Both questions had nine response options ranging from “I do not watch TV, videos or DVDs/I do not use a computer” to “Around seven hours a day or more.” Responses were recorded to illustrate sedentary digital media use as hours. We then computed weighted means for weekly TV and computer use and summed the means together to indicate total sedentary digital media use per hour/day.

##### 2.2.2.5 Covariates

Adolescents’ age and sex were verified through linkage to the National Population Information System at the Population Register Center. The mother's occupation at the time of the child's birth was obtained from the Medical Birth Register maintained by the Finnish Institute for Health and Welfare and used to indicate maternal socioeconomic status (SES) (27). Mothers were categorized as upper-level employees, lower-level employees, manual workers, students, and others. Puberty status at baseline was defined based on self-evaluation of pubertal development using the five-point Tanner scale (28). It consisted of a pictorial assessment of breast development and pubic hair for girls and of pubic hair for boys. We categorized adolescents into prepubertal or pubertal/postpubertal. To account for the baseline situation regarding the adiposity outcomes, BMIz or WHtR at baseline was used as a covariate, respectively.

### 2.3 Statistical analysis

Descriptive statistics were calculated as means and SD, or as counts and percentages (%), and the differences in means or proportions between categories were tested using ANOVA or Chi-squared tests, respectively. By utilizing Cox regression, we computed hazard ratios (HR) with 95% CI to examine the relationship between baseline health behaviors (dietary habits, PA, sleep midpoint, and sedentary digital media use) and the likelihood of belonging to the group with increased BMIz or WHtR compared with being in the decreased or stable groups, considering the time between baseline and follow-up. We conducted crude analyses (model 1) for each health behavior individually; adjusted analyses controlling for baseline age, sex, SES, and baseline BMIz or WHtR, respectively (model 2); and fully adjusted analyses including additionally all other health behaviors (model 3). For the dietary pattern analysis, “healthy eaters” was the reference group, and for meal pattern and breakfast pattern analyses, “regular” was the reference group. We found no collinearity among any variables used in the models. In addition, to consider the role of puberty, we conducted sensitivity analyses using puberty status as an additional covariate in the fully adjusted Cox regression model (*n* = 3,840). All analyses were performed using the SPSS statistical software (version 28.0). Statistical significance was considered as *p* < 0.05.

## 3 Results

### 3.1 Participant characteristics

**Figure 1:**
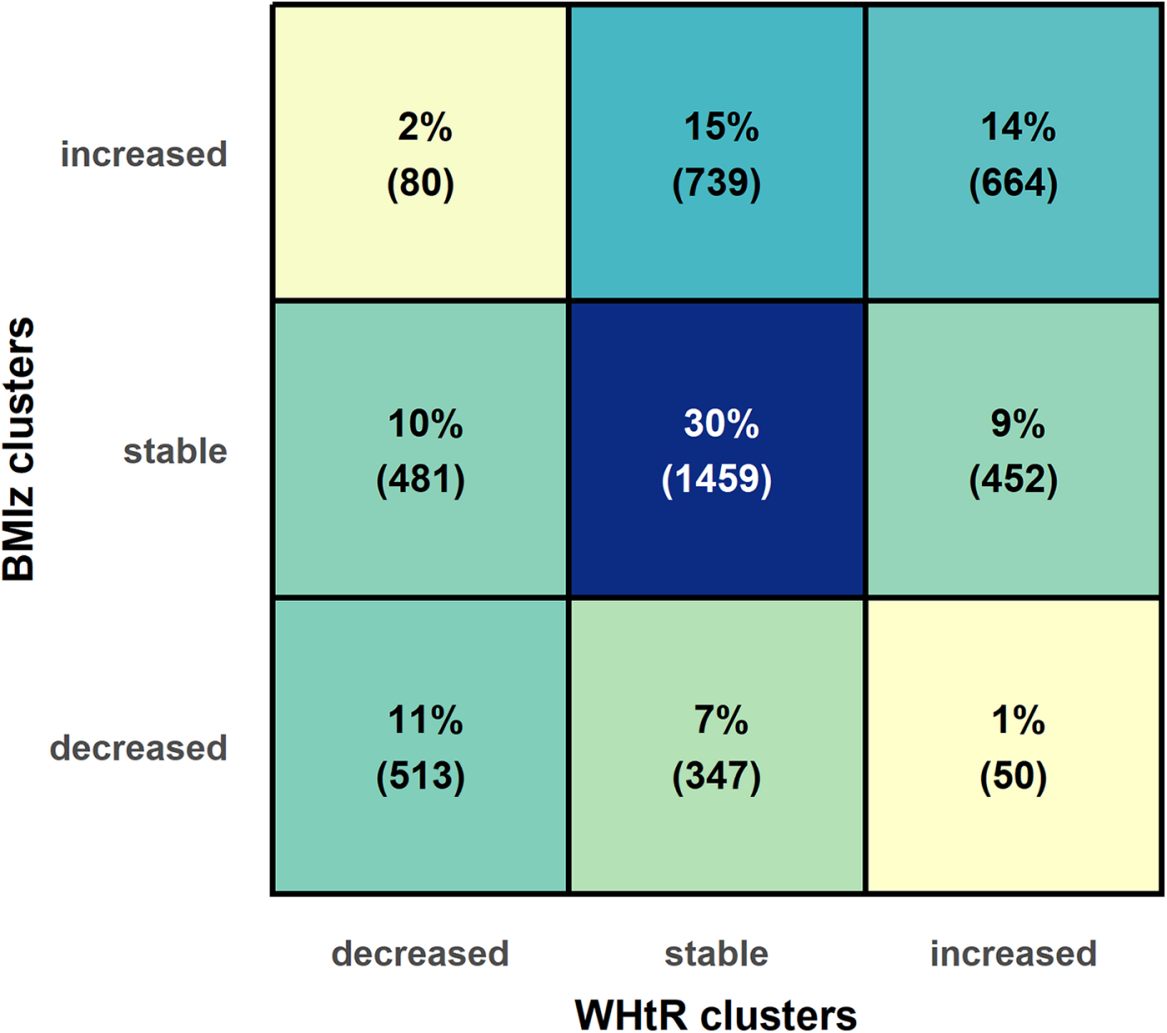
Overlap of BMI *z*-score (BMIz) and waist–height ratio (WHtR) clusters.

The study population consisted of 4,785 Fin-HIT participants (53.0% of girls) whose mean (SD) age was 11.1 (0.8) years at baseline and 13.7 (1.2) years at follow-up (Table 1). The mean follow-up time was 2.6 (0.8) years, ranging from 1.4 to 4.8 years. Of the participants, 910 (19.0%) fell into the decreased BMIz cluster, 2,392 (50.0%) into the stable BMIz cluster, and 1,483 (31.0%) into increased BMIz cluster. For the WHtR clusters, corresponding figures were 1,074 (22.4%), 2,545 (53.2%), and 1,166 (24.4%), respectively. Figure 1 shows the overlap of the BMIz and WHtR clusters. Of the participants, 55.1% were categorized in the corresponding category (e.g., a participant decreased both BMIz and WHtR) and 2.7% in the opposite categories (BMIz decreased but WHtR increased and vice versa).

**Table 1:** Participant characteristics by clusters of change in BMI *z*-score (BMIz) are shown as means (SD), unless otherwise stated.

Participant characteristic	All		Decreased BMIz		Stable BMIz		Increased BMIz		*p* ^a^
	*n* = 4,785		*n* = 910		*n* = 2,392		*n* = 1,483		
Baseline									
Age, y	11.1	(0.8)	11.1	(0.8)	11.0	(0.9)	11.2	(0.8)	<0.001
BMIz	0.17	(0.97)	0.61	(0.96)	0.29	(0.93)	−0.30	(0.86)	<0.001
WHtR	0.43	(0.04)	0.45	(0.05)	0.44	(0.04)	0.42	(0.04)	<0.001
Height, cm	147.5	(8.4)	148.2	(8.2)	147.3	(8.7)	147.4	(8.1)	0.017
Sex, *n* (%)									<0.001
Girl	2,538	(53.0)	443	(48.7)	1,223	(51.1)	872	(58.8)	
Boy	2,247	(47.0)	467	(51.3)	1,169	(48.9)	611	(41.2)	
Weight status, *n* (%)									<0.001
Thin	563	(11.8)	50	(5.5)	197	(8.2)	316	(21.3)	
Normal weight	3,564	(74.5)	627	(68.9)	1,835	(76.7)	1,102	(74.3)	
Overweight	562	(11.7)	199	(21.9)	305	(12.8)	58	(3.9)	
Obese	96	(2.0)	34	(3.7)	55	(2.3)	7	(0.5)	
Central obesity, *n* (%)									<0.001
No	4,408	(92.1)	774	(85.1)	2,196	(91.8)	1,438	(97.0)	
Yes	377	(7.9)	136	(14.9)	196	(8.2)	45	(3.0)	
Puberty status, *n* (%)									0.589
Prepubertal	1,395	(36.3)	277	(37.2)	655	(35.5)	463	(37.0)	
Pubertal/postpubertal	2,445	(63.7)	467	(62.8)	1,190	(64.5)	788	(63.0)	
Missing, *n*	945		744		547		232		
Maternal SES, *n* (%)									0.078
Upper-level employees	1,628	(34.0)	300	(33.0)	838	(35.0)	490	(33.0)	
Lower-level employees	1,928	(40.3)	373	(41.0)	948	(39.6)	607	(40.9)	
Manual workers	472	(9.9)	111	(12.2)	218	(9.1)	143	(9.6)	
Students	444	(9.3)	73	(8.0)	218	(9.1)	153	(10.3)	
Other	313	(6.5)	53	(5.8)	170	(7.1)	90	(6.1)	
Dietary pattern, *n* (%)									0.484
Unhealthy	512	(10.7)	99	(10.9)	243	(10.2)	170	(11.5)	
Vegetable and fruit avoider	2,039	(42.6)	386	(42.4)	1,007	(42.1)	646	(43.6)	
Healthy	2,234	(46.7)	425	(46.7)	1,142	(47.7)	667	(45.0)	
Meal pattern, *n* (%)									0.028
Irregular	1,000	(20.9)	175	(19.2)	481	(20.1)	344	(23.2)	
Regular	3,785	(79.1)	735	(80.8)	1,911	(79.9)	1,139	(76.8)	
Breakfast pattern, *n* (%)									0.805
Irregular	712	(14.9)	140	(15.4)	358	(15.0)	214	(14.4)	
Regular	4,073	(85.1)	770	(84.6)	2,034	(85.0)	1,269	(85.6)	
Physical activity, h/w	6.62	(2.76)	6.30	(2.74)	6.70	(2.77)	6.70	(2.74)	<0.001
Digital media use, h/d	3.17	(2.04)	3.41	(2.07)	3.12	(2.05)	3.10	(2.00)	<0.001
Sleep midpoint, h:mm	2:42	(0:31)	2:41	(0:30)	2:42	(0:32)	2:43	(0:30)	0.388
Sleep duration, h/d	9.68	(0.78)	9.67	(0.78)	9.69	(0.79)	9.68	(0.75)	0.933
Bedtime, h:mm	21:52	(0:46)	21:51	(0:44)	21:52	(0:47)	21:53	(0.44)	0.724
Follow-up									
Age, y	13.7	(1.2)	13.8	(1.3)	13.5	(1.3)	13.8	(1.2)	<0.001
BMIz	0.29	(0.93)	−0.06	(0.97)	0.34	(0.93)	0.44	(0.83)	<0.001
WHtR	0.44	(0.04)	0.43	(0.04)	0.44	(0.05)	0.44	(0.04)	<0.001
Height, cm	162.0	(10.3)	163.7	(10.3)	161.3	(10.5)	162.2	(10.0)	<0.001
Weight status, *n* (%)									<0.001
Thin	368	(7.7)	148	(16.3)	163	(6.8)	57	(3.8)	
Normal weight	3,738	(78.1)	690	(75.8)	1,854	(77.5)	1,194	(80.5)	
Overweight	584	(12.2)	69	(7.6)	317	(13.3)	198	(13.4)	
Obese	95	(2.0)	3	(0.3)	58	(2.4)	34	(2.3)	
Central obesity, *n* (%)									<0.001
No	4,343	(90.8)	855	(94.0)	2,149	(89.8)	1,339	(90.3)	
Yes	442	(9.2)	55	(6.0)	243	(10.2)	144	(9.7)	

BMIz, body mass index *z*-score; SES, socioeconomic status; WHtR, waist–height ratio.

^a^ ANOVA or Chi-square.

### 3.2 Characteristics by BMIz clusters

Table 1 shows the characteristics of the participants by BMIz clusters. The clusters differed by baseline and follow-up age, BMIz, WHtR, height, weight status, central obesity, sex, meal pattern, PA, and digital media use (*p* < 0.05). Moreover, the group with increased BMIz exhibited the lowest mean BMIz value at baseline (*p* < 0.001). Correspondingly, the prevalence of overweight and obesity was 4.4% at baseline but 15.7% at follow-up in the increased BMIz group. At baseline, 3% of the increased BMIz group had central obesity whereas at follow-up, the prevalence was 9.7%. In the decreased BMIz group, the prevalence of overweight and obesity was 25.6% at baseline but 7.9% at follow-up. Similarly, the rate of central obesity decreased from 14.9% to 6.0% during the follow-up period. In the stable BMIz cluster, the proportions of weight status groups were relatively similar at baseline and follow-up. Only minor differences in height were observed between the clusters at any time point.

### 3.3 Characteristics by WHtR clusters

Table 2 shows the participant characteristics by WHtR clusters. The clusters differed by baseline and follow-up age, BMIz, WHtR, height, weight status, central obesity, sex, breakfast pattern, PA, and digital media use (*p* < 0.05). At baseline, the prevalence of central obesity in the increased WHtR group was 7.2%, whereas at follow-up, it was 23.2%. In the decreased WHtR group, the prevalence of central obesity was 18.6% at baseline, whereas at follow-up, it was 5.2%. This was reflected also in the weight status. The lowest proportion of overweight, obesity, and central obesity at baseline and follow-up was in the stable WHtR group. The groups differed in height specifically at follow-up, and the increased WHtR group exhibited the lowest mean height.

**Table 2:** Participant characteristics by clusters of change in waist–height ratio (WHtR) shown as means (SD), unless otherwise stated.

Participant characteristic	All		Decreased WHtR		Stable WHtR		Increased WHtR		*p* ^a^
	*n* = 4,785		*n* = 1,074		*n* = 2,545		*n* = 1,166		
Baseline									
Age, y	11.1	(0.8)	11.2	(0.7)	11.1	(0.9)	11.1	(0.9)	<0.001
BMIz	0.17	(0.97)	0.60	(0.96)	−0.01	(0.91)	0.16	(1.01)	<0.001
WHtR	0.43	(0.04)	0.46	(0.05)	0.42	(0.04)	0.43	(0.05)	<0.001
Height, cm	147.5	(8.4)	148.6	(8.2)	147.0	(8.4)	147.5	(8.7)	<0.001
Sex, *n* (%)									<0.001
Girl	2,538	(53.0)	501	(46.6)	1,364	(53.6)	673	(57.7)	
Boy	2,247	(47.0)	573	(53.4)	1,181	(46.4)	493	(42.3)	
Weight status, *n* (%)									<0.001
Thin	563	(11.8)	59	(5.5)	347	(13.6)	157	(13.5)	
Normal weight	3,564	(74.5)	740	(68.9)	1,981	(77.8)	843	(72.3)	
Overweight	562	(11.7)	236	(22.0)	191	(7.5)	135	(11.6)	
Obese	96	(2.0)	39	(3.6)	26	(1.0)	31	(2.7)	
Central obesity, *n* (%)									<0.001
No	4,408	(92.1)	874	(81.4)	2,452	(96.3)	1,082	(92.8)	
Yes	377	(7.9)	200	(18.6)	93	(3.7)	84	(7.2)	
Puberty status, *n* (%)									0.280
Prepubertal	1,395	(36.3)	327	(36.4)	744	(37.3)	324	(34.2)	
Pubertal/postpubertal	2,445	(63.7)	571	(63.6)	1,252	(62.7)	622	(65.8)	
Missing, *n*	945		176		549		220		
Maternal SES, *n* (%)									0.159
Upper-level employees	1,628	(34.0)	372	(34.6)	879	(34.5)	377	(32.3)	
Lower-level employees	1,928	(40.3)	441	(41.1)	1,024	(40.2)	463	(39.7)	
Manual workers	472	(9.9)	107	(10.0)	227	(8.9)	138	(11.8)	
Students	444	(9.3)	97	(9.0)	240	(9.4)	107	(9.2)	
Other	313	(6.5)	57	(5.3)	175	(6.9)	81	(6.9)	
Dietary pattern, *n* (%)									0.281
Unhealthy	512	(10.7)	114	(10.6)	279	(11.0)	119	(10.2)	
Vegetable and fruit avoider	2,039	(42.6)	467	(43.5)	1,048	(41.2)	524	(44.9)	
Healthy	2,234	(46.7)	493	(45.9)	1,218	(47.9)	523	(44.9)	
Meal pattern, *n* (%)									0.058
Irregular	1,000	(20.9)	208	(19.4)	521	(20.5)	271	(23.2)	
Regular	3,785	(79.1)	866	(80.6)	2,024	(79.5)	895	(76.8)	
Breakfast pattern, *n* (%)									0.039
Irregular	712	(14.9)	165	(15.4)	350	(13.8)	197	(16.9)	
Regular	4,073	(85.1)	909	(84.6)	2,195	(86.2)	969	(83.1)	
Physical activity, h/w	6.62	(2.76)	6.43	(2.75)	6.73	(2.74)	6.55	(2.79)	0.007
Digital media use, h/d	3.17	(2.04)	3.46	(2.04)	3.06	(2.02)	3.15	(2.06)	<0.001
Sleep midpoint, h:mm	2:42	(0:31)	2:42	(0:29)	2:41	(0:31)	2:43	(0:32)	0.395
Sleep duration, h/d	9.68	(0.78)	9.68	(0.74)	9.70	(0.79)	9.66	(0.78)	0.388
Bedtime, h:mm	21:52	(0:46)	21:52	(0:43)	21:51	(0:47)	21:51	(0:46)	0.268
Follow-up									
Age, y	13.7	(1.2)	14.1	(1.2)	13.6	(1.2)	13.5	(1.2)	<0.001
BMIz	0.29	(0.93)	0.29	(0.93)	0.14	(0.88)	0.65	(0.93)	<0.001
WHtR	0.44	(0.04)	0.42	(0.04)	0.43	(0.04)	0.47	(0.05)	<0.001
Height, cm	162.0	(10.3)	165.5	(10.3)	161.4	(10.3)	160.1	(9.8)	<0.001
Weight status, *n* (%)									<0.001
Thin	368	(7.7)	92	(8.6)	240	(9.4)	36	(3.1)	
Normal weight	3,738	(78.1)	835	(77.7)	2,073	(81.5)	830	(71.2)	
Overweight	584	(12.2)	133	(12.4)	201	(7.9)	250	(21.4)	
Obese	95	(2.0)	14	(1.3)	31	(1.2)	50	(4.3)	
Central obesity, *n* (%)									<0.001
No	4,343	(90.8)	1,018	(94.8)	2,429	(95.4)	896	(76.8)	
Yes	442	(9.2)	56	(5.2)	116	(4.6)	270	(23.2)	

BMIz, body mass index *z*-score; SES, socioeconomic status; WHtR, waist–height ratio.

^a^ ANOVA or Chi-square.

### 3.4 Health behaviors and increased BMIz

**Figure 2:**
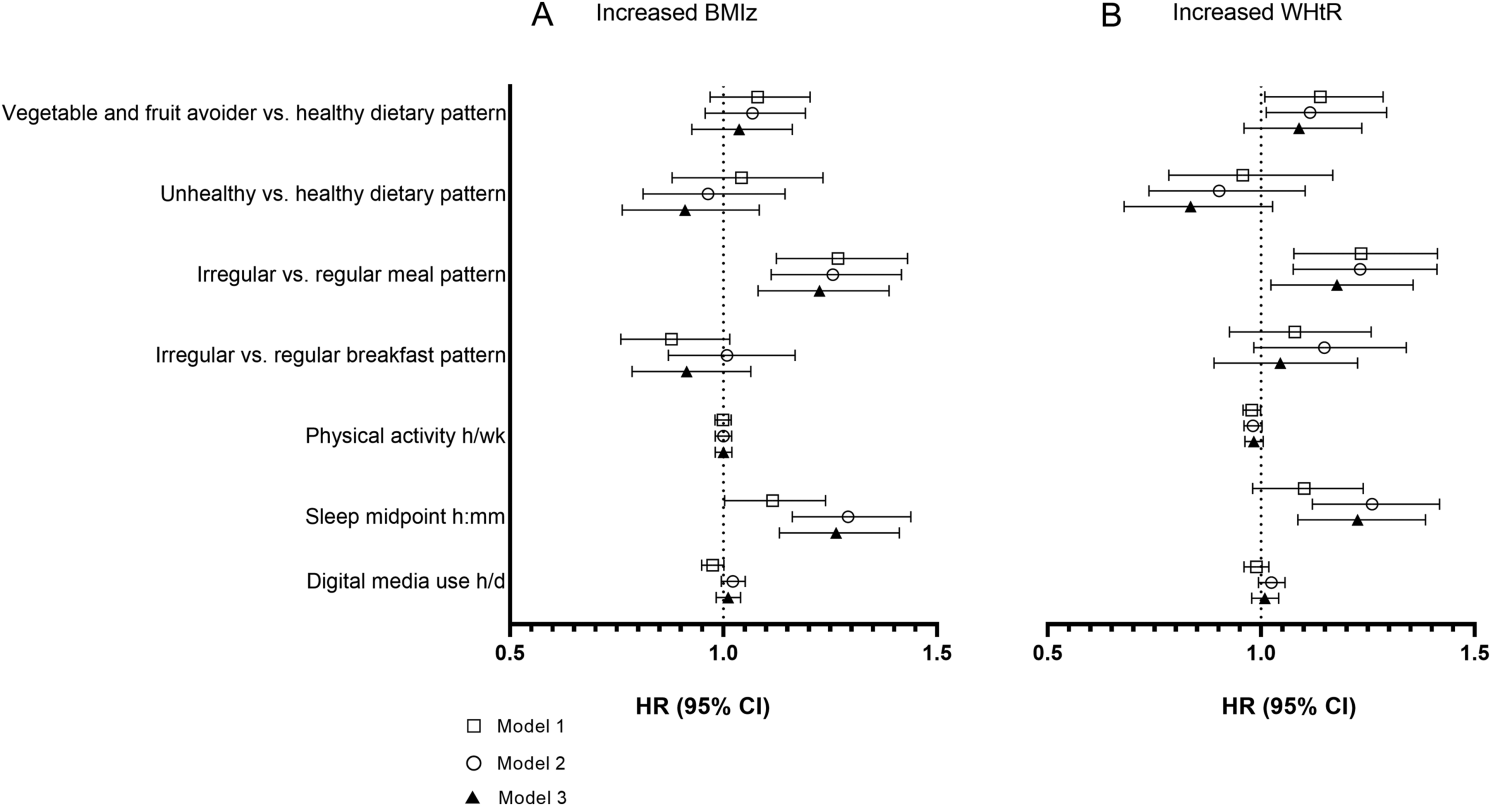
Hazard ratios (HR) with 95% CI for the association between baseline health behaviours and belonging to the group with increased **(A)** BMI *z*-score (BMIz) and **(B)** waist–height ratio (WHtR) compared with others. Model 1, crude; model 2, adjusted for baseline age, sex, maternal SES, and baseline BMIz or WHtR, respectively; model 3, additionally adjusted for all other health behaviors.

Figure 2A shows the association between lifestyle factors and belonging to the increased BMIz group compared with being in the stable or decreased BMIz group from the Cox regression, accounting for the time between baseline and follow-up. Later sleep midpoint was associated with being in the increased BMIz group [fully adjusted model 3: HR 1.26 (95% CI 1.13–1.41)], as did irregular meal pattern [1.23 (1.08–1.39)].

### 3.5 Health behaviors and increased WHtR

Later sleep midpoint associated with increased WHtR in the fully adjusted model [model 3: 1.23 (1.09–1.39); Figure 2B]. Having irregular meal pattern was associated with WHtR increase, as well [1.18 (1.02–1.36)]. In addition, avoiding fruit and vegetables was associated with WHtR increase in models 1 and 2, but the association was no longer significant once all other health behaviors were included in the model.

### 3.6 Sensitivity analyses

We conducted sensitivity analyses using puberty status as an additional covariate in the fully adjusted regression models among those who had information available on puberty status (*n* = 3,840). Adjusting for puberty status verified the findings of the entire sample; later sleep midpoint and irregular meal pattern predicted increased BMIz (1.27 [1.12–1.43]; and 1.17 [1.02–1.34], respectively) and WHtR (1.32 [1.15–1.51]; and 1.22 [1.04–1.42], respectively). No associations emerged for the other health behaviors (data not shown).

## 4 Discussion

This study examined the associations of several health behaviors with an increase in adiposity markers (BMIz and WHtR) during a nearly 3-year follow-up among a large sample of Finnish adolescents. We utilized a data-driven, exploratory method to cluster participants based on changes in BMIz and WHtR and observed that later sleep midpoint and irregular meal pattern were associated independently with increased BMIz and WHtR during a mean 2.6-year follow-up period when all health behaviors were considered in the statistical model. These findings were further validated in sensitivity analyses in those with data available on pubertal status at baseline, implying that the pubertal stage was not affecting the observed associations. To our knowledge, this is the first study to utilize such a clustering method with inspecting simultaneously the effect of dietary habits, PA, sleep, and sedentary digital media use on change in BMIz and WHtR.

Clustering participants based on their BMI and WHtR development provides a different perspective on examining the contributions of health behaviors to adiposity than the traditional BMI categories. In the present study, almost every third child belonged to the cluster of increased BMIz, while every fourth child to the cluster of increased WHtR. Adolescents in the increased BMIz and WHtR groups were on average thinner and had smaller waists at baseline than those with a decreased BMIz and WHtR, who had the highest values at baseline. This was reflected also in the weight status and central obesity. Adolescents in the increased BMIz and WHtR groups were the heaviest and had the largest waists at follow-up. Thus, to account for the starting level, we adjusted the regression analysis for baseline BMIz and WHtR, respectively. In addition, we were concerned that an increase in BMIz might relate to the onset of puberty and not reflect excess adiposity gain. Although BMIz considers the age and sex of the individual, it does not consider the different pubertal growth individuals may have. Moreover, high BMI in early life accelerates linear growth and advances the onset of puberty, while early puberty slows down linear growth during adolescence (29, 30), highlighting that puberty status should be considered when addressing weight gain in adolescence. Therefore, we ran a sensitivity analysis using puberty status as an additional covariate as ∼20% of the adolescents did not have information on puberty status. Adjusting for puberty status yielded similar results as in the entire sample. We acknowledge that we did not have repeated information on pubertal development. Then again, our findings on WHtR resembled those of BMIz. WHtR is a robust measure of abdominal adiposity among children and adolescents and a relatively constant marker of central adiposity regardless of age and sex (31, 32). Moreover, although the mean heights at baseline differed significantly between the clusters, the differences appear negligible. At follow-up, the differences in mean heights between clusters were to some extent more notable, especially according to WHtR clusters; the mean height appeared largest among those in the decreased WHtR group. This is likely explained by the larger proportion of boys in the decreased group. The effect of sex was considered a confounding factor in the regression analysis. Taken together, these suggest that our clusters of increased BMIz and WHtR reflect excessive adiposity gain and not pubertal development. Noteworthy is that the range of change specifically in the group with increased BMIz was rather large; thus, the group included not only those with modest BMIz increase but also those with more excessive gain.

Irregular meal pattern—prevalent in every fifth participant in this sample—is associated with a higher hazard ratio for an increased BMIz and WHtR, suggesting that eating lunch and dinner regularly might be an indication of a healthy eating pattern that could help maintain weight. Indeed, regular eating—that is, breakfast, lunch, and dinner and, if needed, one or two healthy snacks—is recommended as part of healthy eating habits (33). In Finland, a balanced lunch is offered free of charge to all children in elementary and secondary school. Yet not all children enjoy lunch at school, and in our sample, 11% did not eat school lunch every weekday (data not shown), resembling the prevalence in a sample of Finnish sixth graders (∼12 years old) (34). Also, skipping school lunch increases during adolescence (35), and a proper, balanced meal could be substituted with unhealthy snacks. Eating a balanced school lunch was shown to indicate overall healthy eating habits in a Finnish study on adolescents’ eating habits (36), and regular eating pattern mirrors generally healthy eating habits. Family meals are frequently associated with a lower risk of being overweight or obese as well as healthier eating habits (37), which could possibly be relevant to our findings as well. However, we did not have information on who adolescents had dinners with. Regardless, our findings further support the recommendation for regular meals.

Nearly every sixth participant in this study had an irregular breakfast pattern, defined here as consuming breakfast other than a drink less often than every school morning. We decided to investigate breakfast pattern separately from lunch and dinner as breakfast skipping has been previously so clearly associated with the risk of being or becoming overweight or centrally obese among youth (38–40). In contrast with the finding regarding meal pattern, however, breakfast skipping did not associate with increased BMIz or WHtR. Besides linking to weight gain, breakfast skipping appears to be associated with cardiometabolic outcomes such as worsened blood lipid levels, hypertension, and insulin resistance as well as worsened dietary quality (39). During adolescence, the chronotype, that is, an individual's tendency to be active in different intervals during the day (41), tends to shift from an earlier type to a later one (42). Similarly, breakfast skipping has been shown to increase during adolescence (39), perhaps consequent to sleeping later. Taken together, it might be that eating lunch and dinner indicates a healthy dietary behavior that protects from weight gain, and a regular breakfast pattern has a less important role in this age group.

The dietary pattern characteristic of avoiding fruits and vegetables—present in ∼43% of this sample—is associated with increased WHtR in the unadjusted model as well as the model adjusted for background characteristics. However, the association disappeared after adding all the other health behavior variables to the model. No association emerged with the increased BMIz. A whole diet approach examining dietary patterns provides a more holistic view of the habitual diet than examining nutrient intakes or single food items. In general, energy-dense and low-fiber diets associate with later excess weight (43). However, the role of dietary patterns in pediatric obesity or weight gain is not clear (5), and while insufficient fruit and vegetable consumption can influence health adversely, its role in weight gain may not be as relevant as that of other health behaviors, as our results suggest. Moreover, the comparison to different studies is complicated by the fact that dietary patterns achieved by exploratory methods are typically heterogenous (43). In our study, the dietary pattern groups were derived using a short FFQ measuring weekly consumption frequencies of healthy and unhealthy indicatory food items. It is possible that our instrument was not detailed enough to capture important differences in children's eating habits. Moreover, as underreporting can be a serious challenge in dietary studies, we cannot exclude the possibility that underreporting hampered our results.

In this study, later sleep midpoint was associated with increased BMIz and WHtR in the adjusted models. Sufficient sleep is fundamental to health, and sleep can be investigated from several different aspects including duration, quality, consistency, and timing. Short sleep duration is known to be associated with adverse health-related outcomes such as obesity and unhealthy eating habits (44). Sleep timing refers to the placement of sleep within the 24 h of the day and can be measured as sleep onset/offset, bedtime/wake-up time, or midpoint of sleep (45). The association between sleep midpoint and adiposity has been studied to a lesser extent. Sleep timing is a relevant aspect in this age group as a shift towards later sleep timing (eveningness chronotype) tends to happen naturally in adolescence (46). We cannot completely exclude the possibility that the association we observed is partly explained by insufficient sleep duration. Then again, later sleep timing has been linked to excess weight and higher adiposity markers independent of sleep duration in children and adolescents, also among the Fin-HIT participants (24, 45). Indeed, some evidence exists of a positive relationship between later sleep timing and adiposity among children and adolescents, although according to a systematic review, it is still inconsistent (10). Moreover, later sleep timing has been associated with adverse dietary habits, such as breakfast skipping and consumption of unhealthy, energy-dense food, as well as increased sedentary behavior and decreased physical activity (10), providing a plausible mechanism between later sleep midpoint and adiposity gain. In our sample, however, dietary patterns, sedentary digital media use, or physical activity were not associated with belonging to the increased BMIz or WHtR group.

Adolescents in this sample were physically active in their leisure time on average for nearly seven hours a week. The recommendation for this age group is 7 hours of moderate- to vigorous-intensity PA per week (47). The weekly amount of leisure-time PA differed between the clusters, appearing the highest among those in the stable and increased BMIz groups, which could possibly be partially explained due to increases in fat-free mass among those physically active (48). However, the differences in PA hours between clusters were negligible; thus, these results could be due to large sample sizes’ ability to detect very small differences without clinical relevance. Notably, baseline PA did not predict belonging to the clusters of increased BMIz or WHtR in the regression analysis. Previous evidence gathered from longitudinal studies has shown PA to be associated with lower adiposity measures quite consistently later in adolescence, with higher PA intensity having a stronger effect (49). However, our study lacked information on the intensity of the PA, possibly explaining the lack of association.

Adolescents spend on average three hours a day watching TV (including videos and DVSs) and using computers or consoles (for surfing the Internet, for passive gaming, etc). This baseline sedentary digital media use differed between the clusters and appeared highest among those in the decreased BMIz or WHtR group. Then again, these adolescents were also the heaviest at the baseline. However, the baseline digital media use did not predict belonging to the clusters of increased BMIz or WHtR. Previous evidence on the prospective relationship between digital media use and adiposity in childhood and adolescence is scarce and limited, and if associations are found, they are weak or modified by other health behaviors, such as PA (50–52). We did not, however, examine follow-up digital media use or change in digital media use from baseline to follow-up and its associations with changes in adiposity measures, which may have yielded different results. Moreover, the digital media use questions did not include smartphone use as smartphones were not as common when the study began in 2011 as they are now, warranting studies that examine the role of smart device use in adiposity-related outcomes.

Strengths of the study include examining various health behaviors simultaneously, the longitudinal study setting, and the large sample size. In addition to the BMIz, we also used the WHtR as an outcome. BMIz does not differentiate between fat and fat-free mass whereas WHtR is shown to predict both total and trunk adiposity (31). Moreover, it is more sensitive as an early marker of health risks than BMI and detects cardiometabolic risks even among normal-weight children (32, 53). Intra-abdominal adipose tissue is recognized as an active endocrine organ that produces hormones and cytokines, possibly leading to dysregulation of metabolic and inflammatory processes (32). Although feasible in large-scale cohort studies, another limitation is the self-reporting of health behaviors using non-validated questionnaires. However, child reporting at this age is considered more reliable than parental reporting (54). Moreover, retrospective reporting of dietary habits might result in recall biases. To better consider the time between baseline and follow-up measurements, we used Cox regression models in our analyses. We acknowledge that this may have overestimated the effect of time.

This study aimed to predict adiposity gains during a short follow-up with baseline health behaviors. However, adolescence is marked by changes in health behaviors such as increases in soft drink consumption and sedentary behavior as well as decreases in fruit and vegetable consumption and physical activity (55). Thus, we do not know if participants with increased BMIz and WHtR experienced adverse changes in their health behaviors. Limitations of this study also include the participation and drop-out rates and therefore the generalizability of the findings. Of the participants who were invited to the study at baseline recruitment, 36% participated, and of those, 54% continued into the follow-up. Compared with the Finnish population, the Fin-HIT sample has on average higher SES and thus likely healthier lifestyles (56). The prevalence of overweight and obesity is also lower compared with international and national reports (1, 2, 57). Moreover, the participants who continued into the follow-up had higher maternal SES and healthier lifestyle habits than those who did not (56). However, the selective sample does not affect the found associations; on the contrary, the associations would probably be stronger in a more representative sample. We were limited to the mother's occupation at the time of the child's birth as the proxy for SES, although the family's income level, both parents’ occupation, or parental education level may be better indicators.

## 5 Conclusions

In conclusion, our study indicates that adolescents who exhibit a later sleep midpoint and an irregular meal pattern are at a higher risk of adiposity gains. These findings underscore the importance of promoting healthy behaviors such as maintaining an early sleep schedule, obtaining sufficient sleep, and adhering to regular meal pattern, particularly lunch and dinner. Implementing these preventive measures and encouraging adolescents to have early sleep habits and regular meal pattern may help mitigate the development of adiposity among young individuals.

## Data Availability

The raw data supporting the conclusions of this article will be made available by the authors, without undue reservation.
