# Supplementary material for: Irregular meal pattern and later sleep midpoint are associated with increased BMI z-score and waist–height ratio during early adolescence
Source: Front Pediatr. 2024 Nov 15;12:1321024. doi: 10.3389/fped.2024.1321024 (PMC11604410; doi:10.3389/fped.2024.1321024)
Supplement: Supplementary file 1 [file Table1.docx]

**SUPPLEMENTARY MATERIAL**

**Irregular meal pattern and later sleep midpoint are associated with increased BMI z-score and waist–height ratio during early adolescence**

Sohvi Lommi^1,2^, Elina Engberg^1,3^, Aku-Ville Lehtimäki^1^, Reetta Lehto^1,4^ and Heli Viljakainen^1,2*^

^1^ Folkhälsan Research Center, Helsinki, Finland

^2^ Faculty of Medicine, University of Helsinki, Helsinki, Finland

^3^ Department of Psychology and Logopedics, University of Helsinki, Helsinki, Finland

^4^ Department of Food and Nutrition, University of Helsinki, Helsinki, Finland

* Correspondence:

Heli Viljakainen

[heli.viljakainen@helsinki.fi](mailto:heli.viljakainen@helsinki.fi)

**Supplementary material.** Food frequency questionnaire used in the baseline data collection, adapted from the questionnaire used in the WHO Health Behavior in School-aged Children HBSC study

|  | Not at all | Less than once a week | Once a week | 2–4 times a week | 5–6 times a week | Once a day | Several times a day |
| --- | --- | --- | --- | --- | --- | --- | --- |
| Dark bread or ryebread |  |  |  |  |  |  |  |
| Biscuits/cookies |  |  |  |  |  |  |  |
| Sweet pastry (e.g. bun, doughnut, Danish pastry) |  |  |  |  |  |  |  |
| Pizza |  |  |  |  |  |  |  |
| Hamburger or hot dog |  |  |  |  |  |  |  |
| Milk or soured milk |  |  |  |  |  |  |  |
| Ice cream |  |  |  |  |  |  |  |
| Cooked vegetables |  |  |  |  |  |  |  |
| Fresh or grated vegetables, salad |  |  |  |  |  |  |  |
| Fruit or berries |  |  |  |  |  |  |  |
| Juice (unsweetened) |  |  |  |  |  |  |  |
| Sugary juice drink |  |  |  |  |  |  |  |
| Chocolate & sweets |  |  |  |  |  |  |  |
| Salty snacks (e.g. chips, popcorn, salted peanuts) |  |  |  |  |  |  |  |
| Sugary soft drink |  |  |  |  |  |  |  |
| Water |  |  |  |  |  |  |  |
